# The Identification of Novel *CYP2D6* Variants in US Hmong: Results From Genome Sequencing and Clinical Genotyping

**DOI:** 10.3389/fphar.2022.867331

**Published:** 2022-03-21

**Authors:** Ya Feng Wen, Andrea Gaedigk, Erin C. Boone, Wendy Y. Wang, Robert J. Straka

**Affiliations:** ^1^ Department of Experimental and Clinical Pharmacology, College of Pharmacy, University of Minnesota, Twin Cities, MN, United States; ^2^ Division of Clinical Pharmacology, Toxicology and Therapeutic Innovation, Children’s Mercy Research Institute, Kansas City, MO, United States; ^3^ School of Medicine, University of Missouri-Kansas City, Kansas City, MO, United States

**Keywords:** CYP2D6, pharmacogenetics, minority health, population genetics, ethnic variability, targeted exome sequencing, Hmong

## Abstract

**Objective:** Hmong individuals represent a unique East Asian subpopulation in whom limited information concerning pharmacogenetic variation exists. The objectives of this study were to comprehensively characterize the highly polymorphic *CYP2D6* gene in Hmong, estimate allele and phenotype frequencies and to compare results between two testing platforms.

**Methods:** DNA from 48 self-identified Hmong participants were sequenced using a targeted next-generation sequencing (NGS) panel. Star allele calls were made using Astrolabe, manual inspection of NGS variant calls and confirmatory Sanger sequencing. Structural variation was determined by long-range (XL)-PCR and digital droplet PCR (ddPCR). The consensus diplotypes were subsequently translated into phenotype utilizing the activity score system. Clinical grade pharmacogenetic testing was obtained for 12 of the 48 samples enabling an assessment of concordance between the consensus calls and those determined by clinical testing platforms.

**Results:** A total of 13 *CYP2D6* alleles were identified. The most common alleles were CYP2D6*10 and its structural arrangements (37.5%, 36/96) and the *5 gene deletion (13.5%, 13/96). Three novel suballeles (*10.007, *36.004, and *75.002) were also identified. Phenotype frequencies were as follows: ultrarapid metabolizers (4.2%, 2/48), normal metabolizers (41.7%, 20/48) and intermediate metabolizers (52.1%, 25/48); none of the 48 participants were predicted to be poor metabolizers. Concordance of diplotype and phenotype calls between the consensus and clinical testing were 66.7 and 50%, respectively.

**Conclusion:** Our study to explore *CYP2D6* genotypes in the Hmong population suggests that this subpopulation is unique regarding *CYP2D6* allelic variants; also, a higher portion of Hmong participants (50%) are predicted to have an intermediate metabolizer phenotype for CYP2D6 compared to other East Asians which range between 27 and 44%. Results from different testing methods varied considerably. These preliminary findings underscore the importance of thoroughly interrogating unique subpopulations to accurately predict a patient’s *CYP2D6* metabolizer status.

## Introduction

Implementing pharmacogenetic (PGx) testing for clinical practice has been shown to positively impact patient outcomes in a variety of settings and medical conditions (Cavallari et al., 2018; Smith et al., 2019; Hulot et al., 2020). In select cases and healthcare systems, this practice has been demonstrated to be cost-effective (Tanner et al., 2020; Karamperis et al., 2021; Kim et al., 2021; Zhu et al., 2021). However, our ability to develop, validate and utilize clinical guidelines in an equitable manner for all members of society is hindered by the lack of population-specific PGx knowledge. Despite the best efforts from the Association for Molecular Pathology (AMP) Clinical Practice Committee’s Pharmacogenomics Working Group outlining which alleles within the clinically important pharmacogenes should be tested, (Pratt et al., 2018; Pratt et al., 2019; Pratt et al., 2020; Pratt et al., 2021), novel single nucleotide polymorphisms (SNPs) and novel structural variants of clinical relevance have continued to be identified in many less-well-studied populations (Montane Jaime et al., 2013; Boone et al., 2020). This critical gap in knowledge for any sub-population can lead to disparities in health outcomes. This is particularly true for individuals with limited access to health care and those more susceptible to select medical ailments. For example, only until recently, investigators (Hernandez et al., 2014; De et al., 2018) have acknowledged the critical importance to incorporate ethno-specific variants in pharmacogene unique to individuals of African descent that must be included when considering genetic-guided dosing of warfarin (Johnson et al., 2017). Hmong individuals residing in Minnesota represent another example where this under-served and under-resourced population whose life circumstances have led to limited engagement in research, especially research evaluating the safety and efficacy of medications (Park et al., 2018).

In our previous work (Wen et al., 2020) regarding the Very Important Pharmacogenes in the Hmong Community (VIP-Hmong) study, we identified significant differences in allele frequencies between Hmong and East Asians (EA) for 23% (5/22) of actionable genetic variants in eight unique VIPs (*CYP2C9*, *CYP2C19, CYP4F2*, *DYPD*, *G6PD*, *SLC O 1B1*, *TMPT*, *VKORC1*). These differences are even greater between Hmong and Europeans (Wen et al., 2020) and may have clinical consequences when predicting drug dose and response (Sun et al., 2020). For select pharmacogenes, key genetic variants and their frequencies can be predicted with a relatively high degree of confidence across populations. However, for highly polymorphic genes, such as *CYP2D6,* the reliability of pre-designed test panels is limited (Nofziger et al., 2019). This limitation is critical as it impacts our ability to accurately translate PGx knowledge into actionable recommendations for about 21% of important medications including antidepressants, antipsychotics, opioids, and chemotherapeutic agents (Saravanakumar et al., 2019). In addition, *CYP2D6* is highly polymorphic within and between populations which leads to a wide range of potential activity and corresponding predicted therapeutic responses. In the absence of sub-population specific knowledge, the potential for phenotype misclassification due to incomplete genotype assessment is high. Inaccuracies in phenotype prediction can lead to potentially severe adverse drug reactions and/or an altered efficacy due to inappropriate drug selection and dosing.

To characterize the complex *CYP2D6* gene locus for SNPs, small nucleotide insertions/deletions (INDELs), copy number variation (CNVs) including gene deletions and duplications, the presence of gene conversions and structural rearrangements involving the highly similar *CYP2D7* pseudogene, often requires the application of several methods and/or approaches. Methodologies to determine structural variants may include NGS, Sanger sequencing, SNP/indel genotyping (Yang et al., 2017), long-read single molecule real-time (SMRT) sequencing (Qiao et al., 2016; Buermans et al., 2017), and/or allele-specific long-range PCR (ASXL-PCR) (Gaedigk et al., 2015). Additional methods to determine CNVs include TaqMan based assays or droplet digital-PCR (ddPCR) (Gaedigk et al., 2019), allele quantification-based genotyping using Pyrosequencing (Langaee et al., 2015), and multiplex ligation-dependent probe amplification (MLPA) (Qiao et al., 2019). These are either used alone or in combination (Fukunaga et al., 2020). Although there is an increasing number of clinical laboratories within a health care institution offering PGx testing (Krebs and Milani, 2019), such sophisticated methods may not always be available. Commonly, PGx testing is performed in a Clinical Laboratory Improvement Amendments (CLIA)-certified laboratory which includes *CYP2D6* Tier 1 and possibly Tier 2 alleles recommended by AMP on the testing panel. Information about testing companies can be found on the Genetic Testing Registry website, National Center for Biotechnology Information, U.S. National Library of Medicine (National Center for Biotechnology Information, 2022).

In the present study, we utilized XL-PCR and ddPCR to detect CNVs and structural rearrangements (SVs) and Sanger sequencing to resolve and/or validate diplotype calls from NGS data. The first objective of this study was to comprehensively characterize *CYP2D6* genetic variation in a select population of Hmong, determine allele frequencies, and estimate the consequent phenotype frequencies. The second objective was to compare these with those of a clinical-grade test report.

## Materials and Methods

### Study Participants

Genomic DNA from 48 Hmong adults living in either Minnesota or Wisconsin was obtained from two independently conducted studies. Hmong ancestry was based on participants self-reporting that both their parents were of Hmong descent. For the purpose of this present study, 34 samples were selected from the VIP-Hmong study (Wen et al., 2020) and 14 from the Genetics Of hyperUricemia Therapy in Hmong (GOUT-H) study (Roman et al., 2017). All participants provided informed consent and both studies were approved by the University of Minnesota Institutional Review Board (UMN IRB #1702M06041 and IRB #1408M53223, respectively). The GOUT-H study was also registered under clinicaltrial. gov: NCT02371421. To ensure that samples from the two studies represented unrelated participants, the names and date of birth of the participants were cross-checked.

### DNA Isolation

Saliva was collected using ORAgene® DISCOVER kits (OGR-500, DNA Genotek Inc., Ottawa, ON, Canada) and processed per the manufacturer’s protocol. Genomic DNA was extracted using a QIAamp DNA Kit (Qiagen Inc., Germantown, MD, United States). DNA was quantified using a Qubit Fluorometer (Thermo Fisher Scientific, Waltham, MA, United States).

### NGS-Based Targeted Sequencing and Variant Calling

ADMEseq is an NGS-based panel targeting 286 genes involved in drug absorption distribution metabolism and elimination (ADME). Briefly, sequencing was performed using a MiSeq instrument (Illumina, San Diego, CA) with paired end 200 base pair reads to a total data of about 355 MB. The average read depth was about 530X over a panel target of about 660 kilo base pairs. TruSeq Libraries were prepared according to the manufacturer’s protocol: 10 cycles of PCR, followed by enrichment with the ADMEseq custom panel.

The sequence reads were aligned to human genome assembly GRCh37 and variants were detected using the DRAGEN Bio-IT platform v2.5.3 (Illumina, San Diego, CA). Variants were called with positions downsampled to 2000 reads with the following criteria: sequence quality ≥10, mapping quality ≥20, and phred-scaled confidence score ≥20.

### *CYP2D6* Star Allele Calling Using Bioinformatics Tools and Designations of Novel Haplotypes

Astrolabe (v0.8.7.2) (Twist et al., 2016; Twist et al., 2017) was used to call *CYP2D6* star allele diplotypes, which were complemented with experimental CNV analyses described below. Parameters were set based on recommendations by the tool’s authors (see supplementary text for analysis scripts). The BAM and VCF files were used as input for Astrolabe. *CYP2D6* allele designations followed those defined by the PharmVar Consortium (https://www.pharmvar.org) (Gaedigk et al., 2018). All variant calls from Astrolabe were manually inspected and analyzed. Novel haplotypes were fully characterized by XL-PCR and Sanger sequencing as described in the sections below; these were also submitted and subsequently accepted by PharmVar for allele designation.

### Determination of *CYP2D6* Copy Number Variation

XL-PCR was employed to detect structural variants as previously described (Bathum et al., 1998; Gaedigk et al., 2010a; Gaedigk et al., 2010b; Gaedigk et al., 2019). A brief description of each amplicon, its conventional name and size, primer sequences, and cycling conditions are summarized in Supplementary Table S1. All XL-PCR reactions were performed using 2x KAPA LongRange HotStart ReadyMix with dye (Roche Diagnostics). Amplicons were visualized on a 0.7% agarose gel with 1x final concentration of SYBR™ Safe DNA Gel Stain (Thermo Fisher Scientific, MA, United States). Samples with CNVs were confirmed with ddPCR to obtain quantitative CNV information using a QX200 Droplet Digital PCR System (Bio-Rad) as previously described (Gaedigk et al., 2019) with the following modifications: 100 ng of genomic DNA was digested with 8U of *Bam*HI-HF (New England BioLabs, Ipswich, MA) in a 20-µL reaction at 37°C for 1.5 h, followed by 20 min at 65°C for inactivation. Ten-15 ng of digested DNA was run in a multiplex reaction using TaqMan copy number assays targeting the 5′UTR (Hs04078252_cn) and exon 9 (Hs00010001_cn) regions. To achieve optimal cluster separation, the concentration of the exon 9 assay was reduced by half (Whale et al., 2016).

### Sanger Sequencing

Selected samples were Sanger sequenced to resolve ambiguous Astrolabe calls or confirm SNP calls that suggested the presence of a novel haplotype(s) (Gaedigk et al., 2015). See Supplementary Table S1 for more information on primer sequences and ASXL-PCR amplicons.

### Translation of *CYP2D6* Diplotypes Into Phenotype

The activity score (AS) of a *CYP2D6* diplotype was retrieved from the “CYP2D6 allele definition table” and translated into phenotype according to the “*CYP2D6* diplotype-phenotype table” (both available through the PharmGKB at https://www.pharmgkb.org/page/cyp2d6RefMaterials). This method, first described by Gaedigk et al (2008) is recommended by the CPIC and Dutch Pharmacogenetics Working Group (DPWG): AS > 2.25, ultrarapid metabolizer (UM); 1.25 ≤ AS ≤ 2.25, normal metabolizer (NM); 0.25 ≤ AS < 1.25, intermediate metabolizer (IM) and AS = 0, poor metabolizer (PM) (Caudle et al., 2020).

### Clinical Grade *CYP2D6* Pharmacogenetic Testing

Among the 34 participants from the VIP-Hmong study, 12 agreed to participate in focus group discussions pertaining to their perceptions of return of PGx results (Nelson et al., 2020). As such, a commercial RightMed® test from OneOme, LLC was obtained for these participants. The RightMed® test uses quantitative real-time PCR assays to determine copy number at four gene regions, the 3′UTR, intron 2, intron 6, and exon 9 of *CYP2D6*. Variants tested by the RightMed® test panel are listed in Supplementary Table S2.

### Comparison of *CYP2D6* Allele and Phenotype Frequencies With Other East Asian Populations

*CYP2D6* allele frequencies were compared with other EA populations using the “*CYP2D6* frequencies table” available through the PharmGKB at (https://www.pharmgkb.org/page/cyp2d6RefMaterials). CYP2D6 phenotype frequencies for other EA populations, including Chinese, Japanese, Korean, Thai, and Vietnamese were obtained from previous publications (Gaedigk et al., 2017).

### Concordance of *CYP2D6* Diplotypes and Phenotypes Among Methods

*CYP2D6* diplotype and phenotype calls obtained by NGS, CNV analysis and Sanger sequencing (referred to as ‘consensus’ calls) were compared with those provided by the RightMed® clinical test reports. Because of small sample size (*N*=12), only descriptive statistics (count and percentage) were applied.

## Results

### Participant Characteristics

Forty-eight self-identified Hmong participants from VIP-Hmong and GOUT-H were included in the analysis. Demographics of the participants are presented in Table 1.

**TABLE 1:** Characteristics of the 48 Hmong participants from the two independent studies, VIP-Hmong and GOUT-H study.

Characteristics	VIP-Hmong (*N* = 34)	GOUT-H (*N* = 14)	Combined (*N* = 48)
Age (years)	37.1 ± 21.0 (18–84)	41.5 ± 14.4 (25–63)	38.4 ± 19.2 (18–84)
Gender (male)	9 (26.5%)	14 (100%)	23 (47.9%)
Height (inches)	66.7 ± 3.4 (54–67)	63.9 ± 2.7 (59–68)	61.6 ± 3.5 (54–68)
Weight (lbs)	147.9 ± 34.0 (88–231)	192.0 ± 42.4 (126.0–295.8)	160.8 ± 41.5 (88–295.8)
BMI (kg/m^2^)	28.4 ± 6.9 (18.1–53.8)	32.9 ± 5.6 (23.5–45.7)	29.7 ± 6.8 (18.1–53.8)

Continuous variables were reported as mean ± SD (range) and categorical variables were reported as count (percentage). BMI, body mass index; GOUT-H, Genetics Of HyperUricemia Therapy in Hmong Study; SD, standard deviation; VIP-Hmong, Very Important Pharmacogenetics in Hmong study.

### *CYP2D6* Allele and Diplotype Frequencies

**FIGURE 1:**
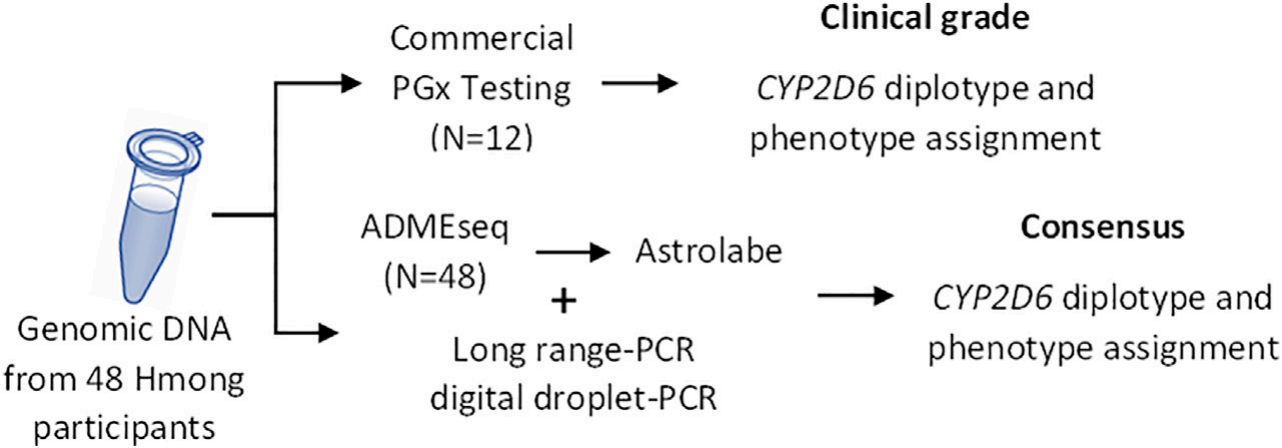
Study Overview. *CYP2D6* diplotype was determined using two different approaches: commercial “clinal-grade” testing and *via* NGS sequencing complemented with CNV analyses (consensus). PGx, pharmacogenetics.

The procedure used to determine *CYP2D6* star allele designations and phenotype calls is summarized in Figure 1.

From the consensus diplotype calls, a total of 13 distinct *CYP2D6* allelic variants were identified (Table 2). The most common star alleles were the decreased function CYP2D6*10 allele (6.3%, 6/96) or structural variants containing this allele, such as CYP2D6*36+*10 (31.3%, 30/96), CYP2D6*36+*10 (REP7) (1.0%, 1/96) and CYP2D6*36×2+*10 (5.2%, 5/96). REP7 indicates that the downstream *CYP2D6* gene has a *CYP2D7*-derived repetitive element including the spacer sequence. Overall, CYP2D6*10 and its structural variants accounted for a total of 43.8% (42/96) of the observed alleles in the Hmong in comparison to allele frequency of 43.6% observed in EAs. The CYP2D6*5 gene deletion (13.5%, 13/96) was the second most common allele in the Hmong in comparison to 4.9% observed in EAs. Other alleles observed in the Hmong included the normal functioning CYP2D6*2 allele (4.2%, 4/96), the decreased function CYP2D6*14 (1%, 1/96) and *41 (1%, 1/96) alleles. The prevalence of CYP2D6*2 in Hmong was less prevalent compared to EA’s where it represented the second most prevalent allele at 12.1%.

**TABLE 2:** *CYP2D6* allele count (frequency) in Hmong compared to East Asians.

*CYP2D6* Alleles^a^	Activity score^b^	Hmong, allele count (%) (*N* = 96)	East Asian^c^, allele % (*N* = 41,366)
*1	1	29 (30.2%)	24.2%
*1x2	2	3 (3.1%)	0.3%
*2	1	4 (4.2%)	12.1%
*5	0	13 (13.5%)	4.9%
*10 ^d^ ^,^ ^e^	0.25	42 (43.8%)	43.6%
*10	0.25	6 (6.3%)	N/A
*36+*10 ^e^	0.25	30 (31.3%)	N/A
*36+*10 (REP7)^f^	0.25	1 (1.0%)	N/A
*36x2+*10	0.25	5 (5.2%)	N/A
*10 (REP7)^f^×2	0.5	1 (1.0%)	0.61%
*14	0.5	1 (1.0%)	0.3%
*36x2	0	1 (1.0%)	1.6%
*41	0.5	1 (1.0%)	2.3%
*75 ^e^	N/A	1 (1.0%)	0.03%

N/A, not available; SNP, single nucleotide polymorphism.

a Allele definitions can be found on the PharmVar CYP2D6 gene page at https://www.pharmvar.org/gene/CYP2D6.

b Activity scores were obtained from the PharmGKB “CYP2D6 allele functionality table”.

c Allele frequencies for East Asian were obtained from the PharmGKB “CYP2D6 frequency table”. The allele frequency of “*10” includes the frequencies of the CYP2D6*10 allele and its structural variations.

d Various structural variations of the CYP2D6*10 allele are listed with indentation.

e Novel suballeles (CYP2D6*10.007, *36.004 and *75.002) were identified in one subject each.

f The downstream region of these CYP2D6*10 alleles contains CYP2D7-like sequences as indicated by “(REP7)”.

Table 3 summarizes all diplotypes identified in this Hmong population cohort and those reported in EAs. The CYP2D6*1/*10 and its structural variant, CYP2D6*1/*36+*10 and CYP2D6*1/*36x2+*10 were the most common diplotypes observed in the Hmong at 18.8% in comparison to 21.1% in an EA population. The second most common diplotype observed in Hmong included CYP2D6*5/*10 (2.1%, 1/48) and its variant CYP2D6*5/*36+*10 (14.6%, 7/48) which was less common in the EA population at 4.2%. In contrast, the second most commonly observed diplotype in EAs is CYP2D6*10/*36+*10 (19%). This compares to a less common frequency in the Hmong for CYP2D6*10/*36+*10 (6.3%) and *36+*10/*36+*10 (8.3%). Supplementary Table S3 provides individual *CYP2D6* diplotype assignments for each of the 48 Hmong subjects.

**TABLE 3:** Comparison of *CYP2D6* diplotype frequencies in 48 Hmong with East Asian populations.

*CYP2D6* diplotype	Activity score^a^	Predicted phenotype^b^	Hmong, N (%) (*N* = 48)	East Asian^c^ (%) (*N* = 20,683)
*1/*1	2	NM	6 (12.5%)	5.8%
*1/*1x2	3	UM	2 (4.2%)	0.2%
*1/*2	2	NM	1 (2.1%)	5.8%
*1/*5	1	IM	4 (8.3%)	2.4%
*1/*10 ^d^ ^,^ ^e^	1.25	NM	9 (18.8%)	21.1%
*1/*10	1.25	NM	1 (2.1%)	N/A
*1/*36+*10	1.25	NM	6 (12.5%)	N/A
*1/*36x2+*10	1.25	NM	2 (4.2%)	N/A
*1/*75.002^e^	Uncertain	Uncertain	1 (2.1%)	0.01%
*1×2/*5	2	NM	1 (2.1%)	N/A
*2/*10	1.25	NM	3 (6.3%)	10.5%
*2/*36+*10 ^e^	1.25	NM	2 (4.2%)	N/A
*2/*36x2+*10	1.25	NM	1 (2.1%)	N/A
*5/*10 ^d^	0.25	IM	8 (16.7%)	4.2%
*5/*10	0.25	IM	1 (2.1%)	N/A
*5/*36+*10	0.25	IM	7 (14.6%)	N/A
*10/*10 ^d^	0.5	IM	10 (20.7%)	19%
*10/*36+*10	0.5	IM	3 (6.3%)	N/A
*36+*10/*36+*10	0.5	IM	4 (8.3%)	N/A
*36+*10/*36x2+*10	0.5	IM	1 (2.1%)	N/A
*36+*10/*36+*10 (REP7)^f^	0.5	IM	1 (2.1%)	N/A
*36x2/*36x2+*10	0.25	IM	1 (2.1%)	NA
*10/*10 (REP7)^f^ *x2*	0.75	IM	1 (2.1%)	N/A
*36+*10/*14	0.75	IM	1 (2.1%)	0.26%
*36+*10/*41	0.75	IM	1 (2.1%)	2.0%

IM, intermediate metabolizer; NM, normal metabolizer; PM, poor metabolizer; UM, ultrarapid metabolizer; N/A, not available.

a Activity sores were calculated from individual genotype presented in Supplementary Table S3 according to the “CYP2D6 allele functionality table” available at PharmGKB.

b Phenotype was determined using activity score and the “CYP2D6 genotype to phenotype translation table” available at PharmGKB.

c Diplotype frequencies for East Asian were obtained from the “CYP2D6 frequency table” available at PharmGKB.

d Various structure variants of *10 is listed below indented.

e Novel suballeles (*10.007, *36.004 and *75.002) were identified in one subject each.

f The downstream region of these CYP2D6*10 alleles is CYP2D7-like as indicated by “(REP7)”.

It is important to note that a few diplotypes with complex structures (4 subjects, Supplementary Table S3) could not be resolved with certainty and assigned the most likely haplotypes to summarize allele and diplotype frequencies. For example, a person with a CYP2D6*36+*10/*36+*10 diplotype could also be *36×2+*10/*10. Similarly, a person with a CYP2D6*36+*10/*36+*10 (REP7) diplotype could be *10[REP7]/*36×2+*10 or *10/*36×2+*10 (REP7). These potential ambiguities in diplotype calling will nonetheless result in the same phenotype prediction regardless of how the gene copies are arranged.

### Identification of Novel *CYP2D6* Suballeles

**FIGURE 2:**
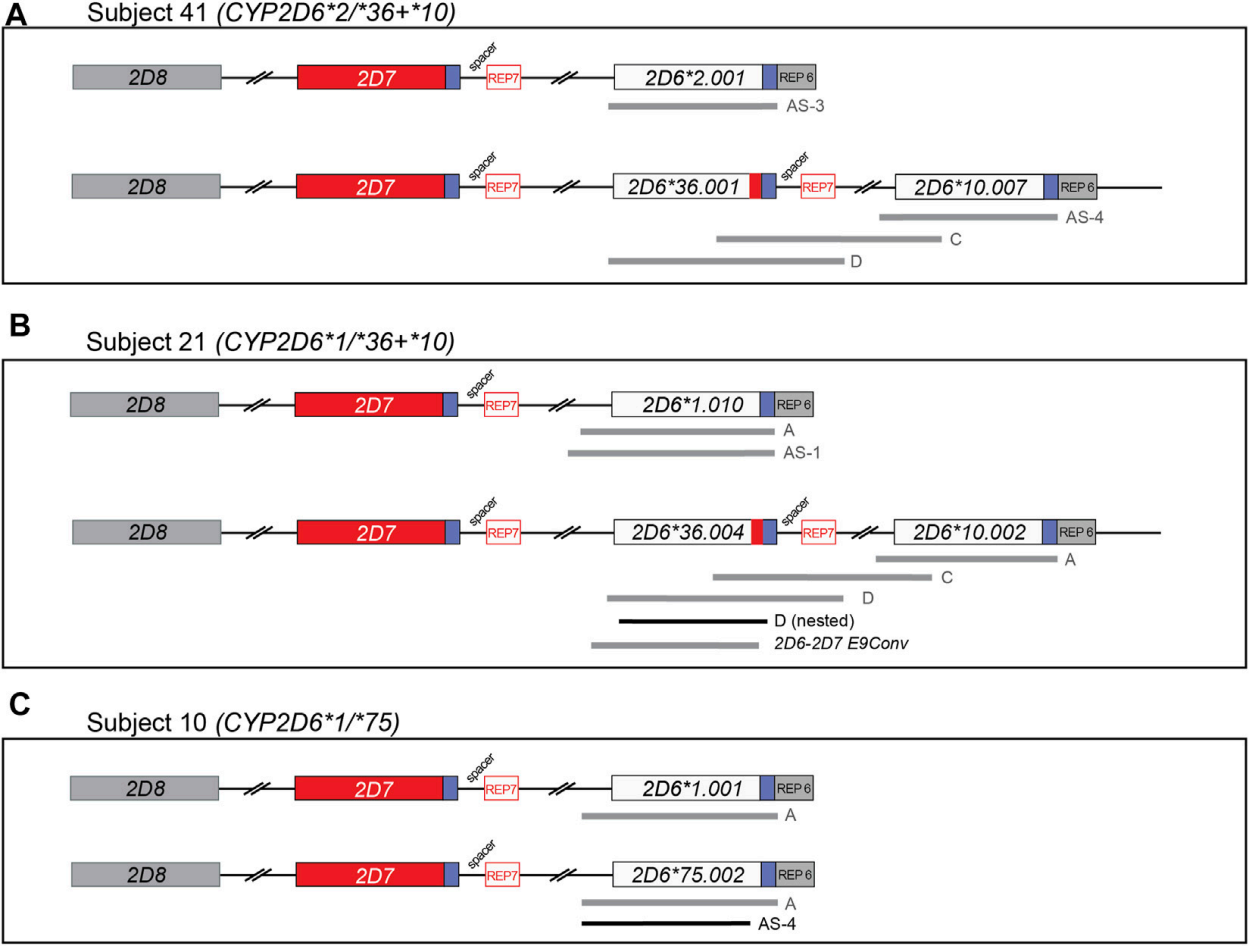
Graphical overview of long-range (XL) PCR fragments generated to characterize gene copy number arrangements and novel allelic variants. Regions amplified from genomic DNA using XL-PCR are represented by gray bars. Selected amplicons were subsequently used as templates to generate sufficient materials for Sanger sequencing (shown in black). Blue boxes downstream of *CYP2D6* and *CYP2D7* denote highly similar regions; those labeled ‘REP’ contain repetitive sequences. *CYP2D7*-derived downstream regions are characterized by the presence of a 1.6 kb “spacer” sequence. See Supplementary Table S1 for a comprehensive list of PCR products generated in this study to characterize the alleles. Panel **(A–C)** describe the summary of XL-PCR products generated to characterize Subject 41, 21, and 10, respectively. All sequence variants found on each of the novel haplotypes are detailed in Figure 3.

**FIGURE 3:**
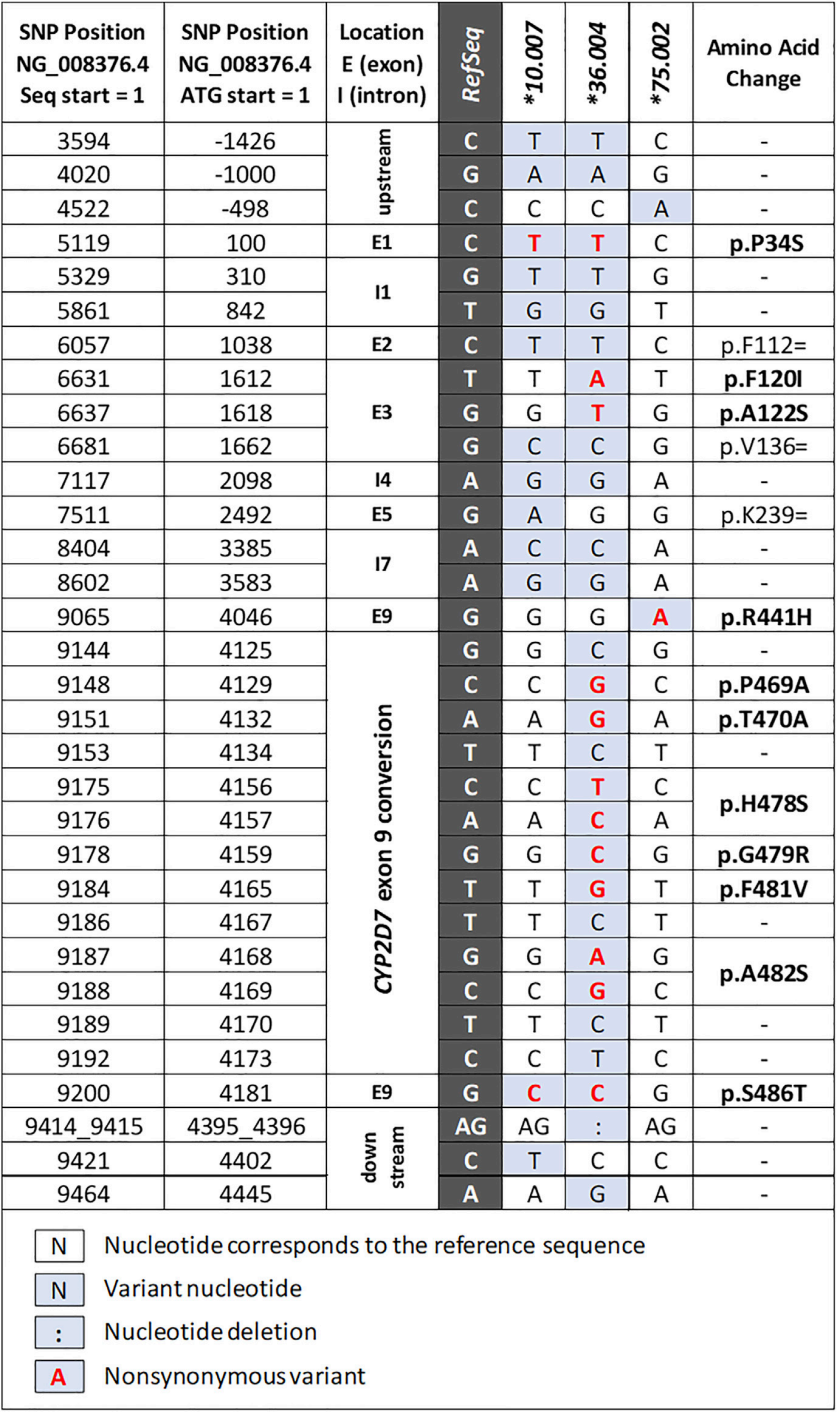
Summary of the novel allele and suballeles identified in the study. The columns to the left provide nucleotide positions counting from the start of the NG_008376.4 reference sequence and the translation start (ATG = +1), respectively. The dark gray column indicates the reference nucleotide of NG_008376.4 which corresponds to the CYP2D6*1.001 allele definition. “Exon 9 conversion” denotes a *CYP2D7-*derived region containing several SNPs (details can be found in the structural variant document available on the PharmVar *CYP2D*6 gene page at https://www.pharmvar.org/gene/CYP2D6).

Three novel suballeles (*10.007, *36.004, and *75.002) were identified in subjects 41, 21, and 10, respectively (Figure 2 and Figure 3). The novel CYP2D6*10.007 suballele was discovered in a CYP2D6*36.001+*10.007 tandem. This structure is supported by the presence of a 10.2 kb long Fragment D, the intergenic fragment C (shown to have 100C>T by genotyping), as well as quantitative ddPCR (5′UTR, 3 copies; exon 9, 2 copies). Sanger sequencing of allele-specific XL-PCR amplicons allowed us to fully characterize the novel suballele and confirm a CYP2D6*2.001 on the other chromosome (Figure 2A and Figure 3, column *10.007) of subject 41. As shown in Figure 2B, subject 21 produced a series of XL-PCR amplicons that together with ddPCR (5′UTR, 3 copies; exon 9, 2 copies) indicated the presence of a CYP2D6*36+*10 tandem. In this case, however, Sanger sequencing of allele-specific amplicons revealed a novel CYP2D6*36 suballele, which was designated *36.004 by PharmVar (Figure 3, column *36.004). The CYP2D6*10 allele was found to match the *10.002 suballele definition, and the allele on the other chromosome was determined to be a CYP2D6*1.010 suballele. The third novel suballele was CYP2D6*75.002 (Figure 3, column *75.002). As illustrated in Figure 2C, Sanger sequencing of an allele specific XL-PCR product allowed us to show that all SNPs initially identified by NGS were on the same chromosome.

### CYP2D6 Phenotype Frequencies

Phenotype was assigned based on the AS for each diplotype (Table 3). In this Hmong population, 4.2% (2/48) were classified as UMs, 41.7% (20/48) as NMs, and 52.1% (25/48) as IMs. No PMs were observed. Phenotype could not be determined for one subject (2.4%). Since function is uncertain for the CYP2D6*75 allele, the CYP2D6*1/*75 diplotype remains ‘undetermined’; however, due to the presence of a CYP2D6*1 allele, this subject is most likely an IM or NM. (Table 4). AS and phenotype assignments for all 48 Hmong participants using different methods are presented in Supplementary Table S3. Comparisons of phenotype distributions between Hmong, EA, and other EA populations are summarized in Table 4. PM, IM and NM phenotype frequencies in the Hmong were similar to those reported for Thai and Vietnamese populations but the Hmong exhibited a higher frequency of UMs compared to the Thai and Vietnamese.

**TABLE 4:** Comparison of *CYP2D6* predicted phenotypes frequencies with other East Asian sub-populations.

Predicted phenotypes	Hmong	Chinese^a^	Japanese^a^	Korean^a^	Thai^a^	Vietnamese^a^	East Asian^b^
UM	4.2	1.1	1.2	1.6	0	0	0.68
NM	43.8	73.3	79.7	64.2	53.7	56.3	51.9
IM	50.0	34.0	27.0	35.0	43.8	43.2	39.2
PM	0	0.4	0.4	0.5	2.5	0.5	0.86
Indeterminate	2.1^c^	—	—	—	—	—	6.7

IM, intermediate metabolizer; NM, normal metabolizer; PM, poor metabolizer; UM, ultrarapid metabolizer. Numbers in the table were presented as percentage.

a Phenotype frequency for East Asian populations was obtained from (Gaedigk et al., 2017).

b Phenotype frequency for East Asian was obtained from “*CYP2D6* frequency table” available at PharmGKB.

c Phenotype is “indeterminate” for the individual with a CYP2D6*1/*75.002 diplotype based on the ‘Genotype to phenotype translation table “available at PharmGKB.

### Concordance of *CYP2D6* Star Alleles and Phenotypes

Concordance for diplotype and phenotype calls between the consensus and clinical (RightMed® test) results was 66.7% (8/12) and 50% (6/12), respectively (Supplementary Table S3).

## Discussion

To our knowledge, this is the first comprehensive characterization of the *CYP2D6* gene locus in Hmong. Although California has the highest population of Hmong, at over 98,000, Minnesota and Wisconsin represent two states which have the second (73,110) and third (49,240) highest populations of Hmong residing in the US (United States Census Bureau, 2016), respectively. There is a growing prevalence of medical conditions within the Hmong community for which the prescribed medications are predominately metabolized by CYP2D6 (Lee et al., 2010; Bart et al., 2012; Lee and Chang, 2012; Lee, 2013; Arcan et al., 2014; Thao et al., 2015). For instance, the Hmong have higher rates of mental health conditions and cervical cancer with lower rates of treatment success (Lee et al., 2010; Lee and Chang, 2012; Lee, 2013; Oyenuga et al., 2018) when compared to general US populations; clinicians have expressed challenges with finding effective medications for the Hmong. The findings from this study indicate that Hmong exhibit distinct differences in allele frequencies and predicted phenotypes relative to other EA populations (Table 4). This implies that the standard medication dose used for patients of European as well as other EA ancestries may not be appropriate for the Hmong patients. This latter comparison is not instinctively intuitive. Knowledge of the unique prevalence or distribution of genetic variations in the Hmong could help clinicians optimize drug therapy (choice of drug and/or dosage), and thus reduce disparities in the quality of treatment while improving health by avoiding predictable, harmful side effects.

In our cohort of Hmong described herein, CYP2D6*10 and its structural variants represented the most common alleles observed at 43.8%. This is consistent with the previously reported frequency of 43.6% in EA populations. However, in the present study, the combined use of quantitative and qualitative CNV analyses enabled us to discriminate between CYP2D6*36+*10 (31.3%) and CYP2D6*10 (6.3%) in the Hmong. It is important to note that the majority of published studies and many commercial tests fail to distinguish between the two. The activity of both alleles is deemed equivalent and therefore, a value of 0.25 is used to calculate the AS of diplotypes containing these alleles. Our observation of the high frequency of the CYP2D6*36+*10 tandem is also consistent with existing data, which suggests that this tandem is exclusively seen in subjects of Asian ancestry including Japanese (Kiyotani et al., 2010), Korean (Kim et al., 2012), and Kinh Vietnamese (Nguyen et al., 2019) populations while the CYP2D6*10 allele on its own is found across populations, but most frequently in EAs (Nofziger et al., 2019). Our findings are also in line with previous studies, which reported almost half of all CYP2D6*10 alleles were found in CYP2D6*36+*10 tandems (Kiyotani et al., 2010; Kim et al., 2012; Qiao et al., 2016; Nguyen et al., 2019). Although it is not necessary to distinguish CYP2D6*10 from CYP2D6*36+*10 for phenotype prediction, the presence of CYP2D6*36+*10 tandems in diplotypes having two or more copies of CYP2D6*10 and/or *36 complicates CNV testing and data interpretation. Thus, it is important to understand which structures are present in a population and at what frequency they occur. This allows for strategic implementation of customized testing approaches to accurately predict CYP2D6 phenotype from genotype data. Additionally, the *CYP2D7*-like REP7 was observed downstream of a CYP2D6*10 allele in two subjects. The presence of the REP7 is likely not impacting the allele’s function, and thus a value of 0.25 was used for AS calculations. Despite not affecting CYP2D6 activity, the observation of the *CYP2D7*-like downstream region with CYP2D6*10 suggests that both hybrid and tandem arrangements could be observed in other alleles within the Hmong population.

Although the sample size of our study was modest, thereby limiting the generalizability of the *CYP2D6* allele and diplotype frequencies to the Hmong community at large, we nonetheless discovered the presence of the CYP2D6*75 allele in this population sample. This allele was first described in Han populations from mainland China (Qin et al., 2008). Notably, the CYP2D6*75 allele found in this study had an additional variant (−498C > A) and was thus designated as a novel suballele (Figure 2) by PharmVar. Moreover, there is not yet enough knowledge regarding the function of this allele; CPIC is currently listing its function as ‘uncertain’. We believe that finding this relatively rare allele in our limited Hmong cohort suggests that there are other “rare alleles” that previously have only been discovered in Chinese or other EA populations which may not only be present in the Hmong but be more prevalent.

We also discovered a novel CYP2D6*10 suballele which has a synonymous SNP in exon 5 (2492G>A; p. K239=). This SNP has not been found in any other haplotype defined by PharmVar and is currently unique to CYP2D6*10.007. The function of this allele is predicted to be equivalent to those of other CYP2D6*10 alleles.

The third novel haplotype identified was designated CYP2D6*36.004. We would like to highlight that this haplotype has several SNPs in addition to 100C>T and the exon 9 conversion which causes the CYP2D6*36 hybrid to be nonfunctional. Due to the presence of these variants, this novel haplotype was designated as a CYP2D6*36 suballele by PharmVar. Additional SNPs observed were: 1612T>A (p.F120I) and 1618G>T (p.A122S), which are the defining core SNPs of CYP2D6*53. Note that 1612T>A is also a core SNP of CYP2D6*49. Due to the presence of 1612T>A and 1618G>T Astrolabe returned a CYP2D6*53 call and CNV analysis suggested the presence of a CYP2D6*36+*10 tandem (the initial tentative diplotype call was CYP2D6*36+*10/*53). However, follow-up studies using XL-PCR and Sanger sequencing revealed that both CYP2D6*53 core SNPs were on the upstream CYP2D6*36 gene copy in the tandem. The diplotype of this subject was ultimately resolved as CYP2D6*1.010/*36.004+*10.002, which can be collapsed to CYP2D6*1/*36+*10 for reporting. First discovered in a Japanese population (Ebisawa et al., 2005), the CYP2D6*53 allele and its activity were found to be comparable to that of CYP2D6*1 (CPIC assigned normal function to CYP2D6*53). Recent studies suggest, however, that CYP2D6*53 may have increased activity based on *in vitro* data (Muroi et al., 2014; Glass et al., 2018). This underscores the necessity to discriminate between CYP2D6*36.004 and *53 when 1612T>A and 1618G>T are detected; these alleles are functionally different (no function vs. normal or possibly increased function). Accurate diplotype determination is even further complicated as commercial tests may only test for 1612T>A (present in CYP2D6*49 and *53) but not 1618G>T (present in CYP2D6*36.004 and *53). This may lead to a false-positive CYP2D6*49 call and decreased function assignment.

Taken together, this study demonstrated the complexity and challenges of accurately calling *CYP2D6* star alleles and diplotypes and corresponding phenotype assignments despite the availability of NGS data and quantitative CNV testing. Additionally, although the CYP2D6 enzyme activity of the three novel suballeles (*10.007, *36.004, and *75.002) are expected to be consistent with their major alleles, a dedicated phenotyping study should be conducted to assert their impact on the phenotype.

The concordance of diplotype calls and respective AS between the consensus calls and the clinical RightMed® test results was 66.7% (8/12). Furthermore, the concordance for predicted phenotypes between the two approaches was only 50% (6/12). The observed discordance was mainly attributed to the differences in technology used between the platforms. The utilization of NGS, XL-PCR and Sanger sequencing allowed us to identify copy number and structural variants and all SNPs present in the individuals. Lower concordance between phenotypes in contrast to diplotype calls can be attributed to using different methods for translating diplotype to phenotype. For example, a diplotype with an AS of 1.25 is categorized as IM to NM by RightMed® test while the CPIC recommended genotype to phenotype translation method categorized this AS as NM. Likewise, an AS of 0.25 is categorized by the RightMed® test as PM to IM while CPIC categorizes this AS as IM. Using different phenotype assignments based on the same genotype can cause confusion for prescribers who are selecting medications based on PGx results.

The increasing number of CPIC guidelines related to CYP2D6 (Crews et al., 2015; Hicks et al., 2015; Bell et al., 2017; Hicks et al., 2017; Goetz et al., 2018) highlights the critical need to understand *CYP2D6* genetic variation and individual activity of this enzyme in special populations, such as the Hmong. This perspective was specifically identified by participants’ engaged in focus group meetings conducted as part of the VIP-Hmong study (Culhane-Pera et al., 2017; Holzer et al., 2020). Specifically themes from focus groups questioning how clinicians decide on drug and dosage selection for Hmong individuals based on guidance and data generated from studies of Western medications (Culhane-Pera et al., 2017) conducted in non-Hmong, raise prescient questions germane to this study. Our study findings could serve to motivate clinicians and payers to adopt and support individualized PGx testing for all individuals.

## Conclusion

Our ‘first in Hmong’ *CYP2D6* study suggests that the Hmong represent a population that exhibit unique CYP*2D6* allelic variation in that a sizable portion of subjects have decreased *CYP2D6* activity. Results utilizing different platforms also illustrated the extent and nature of important sources of variation. These preliminary findings underscore the importance of thoroughly investigating and acknowledging unique populations to have relevant genetic variations which have the potential effect on phenotype prediction accuracy, therapeutic drug response and possibly important clinical outcomes.

## Data Availability

The datasets presented in this study can be found in online repositories. The names of the repository/repositories and accession number(s) can be found below: https://www.pharmvar.org/haplotype/1720, https://www.pharmvar.org/haplotype/1725, https://www.pharmvar.org/haplotype/1728.
