# Supplementary material for: The Identification of Novel CYP2D6 Variants in US Hmong: Results From Genome Sequencing and Clinical Genotyping
Source: Front Pharmacol. 2022 Mar 21;13:867331. doi: 10.3389/fphar.2022.867331 (PMC8979107; doi:10.3389/fphar.2022.867331)
Supplement: Supplementary file 1 [file DataSheet1.docx]

Supplementary Material

**Supplementary Table 1.** Primers and their product sizes for *CYP2D6* copy number variation determination.

| **XL-PCR** | **Primer Sequence (5'-3')** | **Annealing  Temp. (°C)** | **Amplicon (kb)** | **Description** |
| --- | --- | --- | --- | --- |
| Fragment A | F: TCACCCCCAGCGGACTTATCAACC | 68 | 6.7 | Universal primers amplify the entire *CYP2D6* gene |
|  | R: CGACTGAGCCCTGGGAGGTAGGTAG |  |  |  |
| Fragment B | F: CCATGGAAGCCCAGGACTGAGC | 68 | 3.5 | *CYP2D6* duplication (specific for intergenic region) |
|  | R: CGGCAGTGGTCAGCTAATGAC |  |  |  |
| Fragment C | F: AGGAGGCAAGAAGGAGTGTCAGG | 68 | 10.5 | Amplifies from intron 6 of the duplicated gene with a *CYP2D6*-like REP-DUP region through intron 2 of the downstream gene |
|  | R: CCGGATTCCAGCTGGGAAATGCG |  | 12.1 | Amplifies from intron 6 of the duplicated gene with a CYP2D7-like REP-DUP region through intron 2 of the downstream gene |
| [Fragment E] | F: AGGAGGCAAGAAGGAGTGTCAGG | 68 | 4.9 | Amplifies *CYP2D6* intron 6 of the duplicated gene past a *CYP2D6*-like REP-DUP region |
|  | R: CCTGTAGTGTCAGTGACTCAGAAGGCTG |  | 6.5 | Amplifies *CYP2D6* intron 6 of the duplicated gene past a *CYP2D7*-like REP-DUP region |
| Fragment H | F: TCCGACCAGGCCTTTCTACCAC | 68 | 5.0 | Amplifies *CYP2D7-CYP2D6* hybrid genes |
|  | R: CGACTGAGCCCTGGGAGGTAGGTAG |  |  |  |
| Fragment D | F: CCAGAAGGCTTTGCAGGCTTCAG | 68 | 10.2 | Amplifies entire duplicated gene with a *CYP2D7*-like REP-DUP region (e.g., **36* of a **36+*10* tandem) |
|  | R: CGGCAGTGGTCAGCTAATGAC |  | 8.6 | Amplifies entire duplicated gene with a *CYP2D6*-like REP-DUP region (e.g., **1x2, *2x2*) |
| Fragment D (nested) | F: CAAAGGCCATCATCAGCTCC | 63 | 7.3 | Nested PCR using 1:2000 dilutions of Fragment D as a template |
|  | R: CCTTCTGACTGACTCGGTGCC |  |  |  |
| *5 Gene Deletion | F: CTCCAGCCTCCACCAGTCCAG | 68 | 2.9 | Amplifies from the *CYP2D7*-spacer to downstream of the *CYP2D6* locus (e.g., **5* gene deletion) |
|  | R: CAGGCATGAGCTAAGGCACCCAGAC |  |  |  |
| [Alternative *5] | F: AGCCACTCTCGTGTCGTCAGCTT | 68 | 5.0 | Amplifies *CYP2D7* exon 9 to downstream of the *CYP2D6* locus (e.g., **5* gene deletion or gene copy positioned at the 3' end of the gene locus with a *CYP2D7*-like REP region) |
|  | R: CAGGCATGAGCTAAGGCACCCAGAC |  | 3.0 | Amplifies *CYP2D7* exon 9 to downstream of the *CYP2D6* locus (e.g., **36* singleton with a *CYP2D6*-like REP region) |
| CYP2D6-REP7 XL-PCR | F: GCCACCATGGTGTCTTTGCTTTCCTGG | 70 | 3.6 | Amplifies *CYP2D6* exon 9 to downstream of gene locus with a *CYP2D6*-like REP region |
|  | R: CAGGCATGAGCTAAGGCACCCAGAC |  | 5.3 | Amplifies *CYP2D6* exon 9 to downstream of gene locus with *CYP2D7*-like REP region |
| 2D6-2D7E9Conv | F: TCACCCCCAGCGGACTTATCAACC | 63 | 6.1 | Amplifies from upstream CYP2D6 (universal forward primer) through a *CYP2D*7-derived exon 9 (e.g., **36*) |
|  | R: GGGGTCACCAGAAAGCTG |  |  |  |
| internal control amplicon | F: GCATGCACAGCTCAGCACTGC | 68 | 3.8 | Amplifies the IL-10 gene as an internal control (used in select XL-PCR reactions) |
|  | R: GCCACCCTGATGTCTCAGTTTCG |  |  |  |
| AS-1 | F: GAGGCAACCTGCTCGG**G** | 63 | 6.9 | Allele-specific XL-PCR using a forward primer to amplify the -2178G allele |
| AS-2 | F: CCTGGACAACTTGGAAGAACC**C** | 65 | 6.3 | Allele-specific XL-PCR using a forward primer to amplify the **-**1584C allele |
| AS-3 | F: CCTGGACAACTTGGAAGAACC**G** | 70 | 6.3 | Allele-specific XL-PCR using a forward primer to amplify the -1584G allele |
| AS-4 | R: GCTCCCCGAGGCATGgA**T** | 63 | 5.9 | Allele-specific XL-PCR using a reverse primer to amplify the 4046A allele |

XL-PCR, long-range PCR; XL-PCR shown in brackets “[ ]” were duplexed with an internal amplification control

**Supplementary Table 2.** *CYP2D6* single nucleotide polymorphisms (SNPs) tested by the commercial pharmacogenetic testing company (RightMed® test from OneOme, LLC).

| **SNP position NG_008376.4 ATG start = 1** | **SNP rs IDs** | **Variant  (SNV or structural)** | **Star alleles reported^1^** |
| --- | --- | --- | --- |
| -1584 | rs1080985 | C>G | ****2A****, *11, *14, *31, *35, *63* |
| 31 | rs769258 | G>A | **35* |
| 100 | **rs1065852** | C>T | ****4, *4J, *4N****, *10, *36, *64, *68, *69, *114* |
| 124 | rs5030862 | G>A | **12* |
| 137 | rs774671100 | insT | **13, *15* |
| 882 | rs201377835 | G>C | **11* |
| 1022 | **rs28371706** | C>T | ****17****, *64* |
| 1708 | **rs5030656** | delT | ****9****, *109* |
| 1759 | rs5030865 | G>T | **8* |
| 1759 | rs5030865 | G>A | **14, *114* |
| 1847 | **rs3892097** | G>A | ****4, *4J, *4M, *4N*** |
| 2540 | rs72549353 | delAACT | **19* |
| 2550 | **rs35742686** | delA | ****3*** |
| 2616 | **rs5030655** | delAAG | ****6****,* ****6C*** |
| 2851 | **rs16947** | C>T | ****2, *2A****, *8, *11, *12, *14,* ****17****, *19, *29, *31, *35,* ****41****, *42, *63, *69, *91, *114* |
| 2936 | rs5030867 | A>C | **7* |
| 2989 | **rs28371725** | G>A | ****41****, *69, *91* |
| 3184 | **rs59421388** | G>A | ****29****, *70, *109* |
| 3260 | rs72549346 | insGT | **42* |
| 4043 | rs267608319 | G>A | **31* |
| 4134 | rs765776661 | insGTGCCCACT | **18* |
| 4181 | **rs1135840** | G>C | ****2, *2A, *4, *4N****,* ****6C****, *8,* ****10****, *11, *12, *14,* ****17****, *19,* ****29****, *31, *35 *36,* ****41****, *42, *64, *69, *70, *114* |
| 7959 | rs79292917 | G>A | **59* |

Bolded and underscored star alleles are the Tier 1 and Tier 2 variant alleles recommended for testing by the Association for Molecular Pathology ([Pratt et al., 2021](#_ENREF_42)). Tested SNP rs IDs and reported star alleles were obtained from OneOme Gene details: Additional test information at https://oneome.com/gene-details/ (Accessed October 5, 2021). SNP positions and variant definitions were based on information on PharmVar *CYP2D*6 gene page at <https://www.pharmvar.org/gene/CYP2D6> (Accessed October 5, 2021).

^1^The delta-delta Ct (ddCt) method was used to quantify *CYP2D6* copy number relative to RNaseP, which is always present in two copies. For duplicated and multiplied alleles, the variant to wild-type ratios of any variants present was used to identify which allele has been duplicated. Gene deletion was detected using PCR with allele-specific hybridization (personal communication with the scientists at OneOme, LLC).

**Supplementary Table 3.** *CYP2D6* diplotype assignment, activity score, and predicted phenotype using consensus and commercial ‘clinal-grade’ testing approach for 48 Hmong participants.

|  | **Consensus** | | | **Commercial** | | |
| --- | --- | --- | --- | --- | --- | --- |
| **ID** | **Diplotype** | **AS^1^** | **Phenotype^2^** | **Diplotype** | **AS^1^** | **Phenotype^2^** |
| 1 | **36*+**10/*36*+**10[REP7]^3^* | 0.5 | IM |  |  |  |
| 2 | **1/*1x2* | 3 | UM |  |  |  |
| 3 | **2/*36*+**10* | 1.25 | NM | **2A/*10x2* | 1.5 | NM |
| 4 | **1/*1* | 2 | NM |  |  |  |
| 5 | **1/*36*+**10* | 1.25 | NM | **1/*36+*10* | 1.25 | IM to NM |
| 6 | **36*+**10/*41* | 0.75 | IM | **36+*10/*41* | 0.75 | IM |
| 7 | **10/*36*+**10* | 0.5 | IM | **10/*36+*10* | 0.5 | IM |
| 8 | **1/*36*+**10* | 1.25 | NM |  |  |  |
| 9 | **1/*2* | 2 | NM |  |  |  |
| 10 | **1.001/*****75.002*** | Indeterminate | Indeterminate |  |  |  |
| 11 | **1/*5* | 1 | IM | **1/*5* | 1 | IM |
| 12 | **5/*36*+**10* | 0.25 | IM | **5/*36+*10* | 0.25 | PM to IM |
| 13 | **1/*36*+**10* | 1.25 | NM | **1/*36+*10* | 1.25 | IM to NM |
| 14 | **36x2/*36x2*+**10^3^* | 0.25 | IM | **5/*36xN+*10* | 0.25 | PM to IM |
| 15 | **1x2/*5* | 2 | NM | **1/*5* | 1 | IM |
| 16 | **1/*1* | 2 | NM |  |  |  |
| 17 | **5/*36*+**10* | 0.25 | IM |  |  |  |
| 18 | **10/*36*+**10* | 0.5 | IM |  |  |  |
| 19 | **1/*10* | 1.25 | NM |  |  |  |
| 20 | **36*+**10/*36x2*+**10^3^* | 0.5 | IM |  |  |  |
| 21 | **1.010/*****36.004***+ **10.002* | 1.25 | NM | **1/*36+*10* | 1.25 | IM to NM |
| 22 | **1/*5* | 1 | IM |  |  |  |
| 23 | **36*+**10/*36*+**10^3^* | 0.5 | IM |  |  |  |
| 24 | **14/*36*+**10* | 0.75 | IM | **14/*36+*10* | 0.75 | IM |
| 25 | **10[REP7]x2/*10* | 0.75 | IM |  |  |  |
| 26 | **36*+**10/*36*+**10^3^* | 0.5 | IM |  |  |  |
| 27 | **10/*36*+**10* | 0.5 | IM |  |  |  |
| 28 | **5/*10* | 0.5 | IM |  |  |  |
| 29 | **1/*36*+**10* | 1.25 | NM |  |  |  |
| 30 | **5/*36*+**10* | 0.25 | IM |  |  |  |
| 31 | **5/*36*+**10* | 0.25 | IM |  |  |  |
| 32 | **1/*1x2* | 3 | UM |  |  |  |
| 33 | **5/*36+*10* | 0.25 | IM |  |  |  |
| 34 | **36*+**10/*36*+**10^3^* | 0.5 | IM | **36+*10/*36+*10* | 0.5 | IM |
| 35 | **1/*36*+**10* | 1.25 | NM |  |  |  |
| 36 | **5/*36*+**10* | 0.25 | IM |  |  |  |
| 37 | **5/*36*+**10* | 0.25 | IM |  |  |  |
| 38 | **1/*5* | 1 | IM |  |  |  |
| 39 | **36*+**10/*36*+**10^3^* | 0.5 | IM |  |  |  |
| 40 | **1/*1* | 2 | NM |  |  |  |
| 41 | **2.001/*36*+****10.007*** | 1.25 | NM |  |  |  |
| 42 | **1/*36x2*+**10* | 1.25 | NM |  |  |  |
| 43 | **1/*5* | 1 | IM |  |  |  |
| 44 | **1/*1* | 2 | NM |  |  |  |
| 45 | **1/*1* | 2 | NM |  |  |  |
| 46 | **1/*1* | 2 | NM |  |  |  |
| 47 | **2/*36x2*+**10* | 1.25 | NM |  |  |  |
| 48 | **1/*36x2*+**10* | 1.25 | NM |  |  |  |

AS, activity score; ddPCR, digital droplet PCR; IM, intermediate metabolizer; NM, normal metabolizer; PM, poor metabolizer; UM, ultrarapid metabolizer; XL-PCR, long-range PCR. Bolded alleles indicate novel haplotypes (star alleles) identified in the study.

^1^Activity scores were calculated from individual diplotypes presented in Supplemental Table 3 according to the “*CYP2D6* allele functionality table” available at PharmGKB.

^2^Phenotype for the consensus calls were determined per the “*CYP2D6* diplotype-phenotype table” available at PharmGKB. Phenotypes for the commercial test are shown as reported.

^3^These diplotypes could not be resolved with certainty and the most likely diplotype is presented.

**Supplementary Text**. Analysis scripts for *CYP2D6* star allele calling using Astrolabe
